# Elevated SH3 and Multiple Ankyrin Repeat Domains 2 Expression Correlates With Improved Glioma Prognosis

**DOI:** 10.1155/2024/6565925

**Published:** 2024-10-04

**Authors:** Wenlin Li, Haiping Shi, Jimin He

**Affiliations:** Department of Neurosurgery, Suining Central Hospital, Suining, Sichuan, China

**Keywords:** biomarkers, glioma, neuro-oncology, prognosis, SHANK2, survival analysis

## Abstract

This study investigates the prognostic significance of SH3 and multiple ankyrin repeat domains 2 (SHANK2) gene expression in glioma patients, using data from The Cancer Genome Atlas (TCGA), the Genotype-Tissue Expression (GTEx) project, and the Gene Expression Omnibus (GEO). Through comprehensive analysis, we found a significant association between higher SHANK2 expression and improved survival outcomes across various glioma subtypes. To further validate the clinical relevance of SHANK2, we conducted cellular experiments involving siRNA-mediated knockdown of SHANK2 in U87 and A172 glioma cell lines. Quantitative real-time PCR (qPCR) and Western blot analyses confirmed the successful knockdown of SHANK2, and subsequent MTT assays revealed that silencing SHANK2 significantly promoted glioma cell proliferation. These findings underscore the potential role of SHANK2 as a tumor suppressor in glioma. The study also introduces a multivariate prognostic model incorporating SHANK2, providing a novel perspective on glioma prognosis. While the retrospective nature of the study presents limitations, our results suggest that SHANK2 expression could serve as a valuable biomarker for glioma prognosis and inform future therapeutic strategies.

## 1. Introduction

Gliomas represent a complex and heterogeneous group of primary brain tumors, arising from glial cells that support and protect neurons in the central nervous system [1]. Characterized by their highly invasive nature and challenging prognosis, gliomas are classified based on their cell of origin, including astrocytomas, oligodendrogliomas, and ependymomas, among others. The World Health Organization (WHO) further stratifies these tumors into Grades I through IV, reflecting a spectrum from low-grade gliomas (LGGs), which are slower growing and more amenable to treatment, to high-grade gliomas (HGGs), such as glioblastoma multiforme (GBM), known for their aggressive behavior and poor outcomes [2].

The prevalence of gliomas varies globally, but glioblastoma remains the most common and deadliest form, accounting for approximately 45% of all primary malignant brain tumors. Despite advances in neuro-oncology, the incidence of gliomas has shown a slight increase in recent decades, with environmental, genetic, and lifestyle factors contributing to individual risk [3].

Current treatment strategies for gliomas are multifaceted, aiming to maximize patient survival while preserving quality of life. Surgical resection is often the first line of treatment, especially for accessible tumors with well-defined margins. However, the infiltrative nature of gliomas frequently makes complete removal challenging. Adjuvant therapies, including radiotherapy and chemotherapy (notably temozolomide for HGGs), are standard. Recent years have seen the emergence of targeted therapies and immunotherapies, though their effectiveness varies and is the subject of ongoing research. Treatment is further personalized through the use of molecular profiling, enabling more precise targeting of the tumor's genetic and epigenetic landscape. Despite these advancements, the prognosis for glioma patients, particularly those with high-grade tumors, remains dismal, with median survival times for GBM patients hovering around 15 months postdiagnosis [4]. This stark reality underscores the urgent need for novel biomarkers that can predict disease progression and response to therapies, paving the way for more targeted and effective treatment paradigms [5].

The SH3 And Multiple Ankyrin Repeat Domains 2 (SHANK2) gene, known for its role in synaptic architecture and function [6–8], has recently attracted attention for its potential involvement in cancer [9]. Although research on SHANK2 in cancer is still emerging, studies in various malignancies suggest that it may play a role in tumor development and progression. For instance, in renal cell carcinoma, SHANK2 expression has been linked to tumor growth and metastasis, indicating its potential as a biomarker for aggressive disease [10]. Similarly, in breast cancer, alterations in SHANK2 expression have been associated with changes in cellular adhesion and migration, implicating its involvement in tumor invasiveness [11].

As we delve into the potential role of the SHANK2 gene in glioma prognosis, it is imperative to understand the foundation upon which this research stands. Gliomas, with their diverse pathological features and complex treatment landscapes, present a formidable challenge in neuro-oncology. Identifying genetic factors that can influence prognosis offers a promising avenue for advancing our understanding and management of this devastating disease.

## 2. Methods

### 2.1. Study Population and Data Acquisition

Data for this retrospective study were obtained from publicly accessible databases: The Cancer Genome Atlas (TCGA), the Genotype-Tissue Expression (GTEx) project, and the Gene Expression Omnibus (GEO). These databases provided comprehensive clinical information, gene expression profiles, and follow-up data for glioma patients and corresponding normal tissue samples.

### 2.2. Gene Expression Quantification

SHANK2 gene expression data were extracted from the aforementioned databases. For TCGA and GEO datasets, normalization of the raw data was performed using standardized pipelines to enable cross-comparison.

### 2.3. Statistical Analysis

Survival analysis was performed using the Kaplan–Meier estimates and Cox proportional hazards regression models. Multivariate models were adjusted for known prognostic factors including age, gender, histological type, IDH mutation status, and 1p/19q codeletion status. Hazard ratios (HRs) and 95% confidence intervals (CIs) were calculated for the assessment of SHANK2's prognostic value.

### 2.4. Model Development and Validation

A prognostic nomogram was constructed from the final multivariate model to predict individualized 1-, 2-, and 3-year survival probabilities. The performance of the nomogram was validated through calibration plots to assess its accuracy and discriminative ability. Statistical computations were conducted with R software, and a *p* value < 0.05 was deemed significant for all tests.

### 2.5. Cell Culture and Transfection

U87 and A172 glioma cell lines were cultured in Dulbecco's Modified Eagle Medium (DMEM) supplemented with 10% fetal bovine serum (FBS), 100 U/mL penicillin, and 100 *μ*g/mL streptomycin (Gibco). Cells were maintained in a humidified incubator at 37°C with 5% CO2. For siRNA transfection, cells were seeded in six-well plates and allowed to reach 60%–70% confluency. siRNA targeting SHANK2 (No. 268302 and No. 136495, from Thermo Fisher Scientific) or a nontargeting control siRNA (Cat. 4404021 from Thermo Fisher Scientific) was transfected into the cells using Lipofectamine RNAiMAX (Invitrogen) according to the manufacturer's protocol [12]. After 48 h, cells were harvested for subsequent analyses to assess the efficiency of SHANK2 knockdown and its effects on cell proliferation.

### 2.6. Quantitative Real-Time PCR (qPCR)

Total RNA was isolated from U87 and A172 cells using the RNAqueous-Micro Kit (Invitrogen) in accordance with the manufacturer's protocol. Reverse transcription of the extracted RNA was carried out with the SuperScript VILO cDNA Synthesis Kit (Invitrogen). qPCR was performed using the SYBR Green Lo-Rox Fast Mix (Bioline) on the ABI 7500 Fast Real-Time PCR system (Applied Biosystems). Each sample was run in triplicate. Relative mRNA levels were determined using the relative standard curve method, with normalization to the average expression of the reference gene glyceraldehyde 3-phosphate dehydrogenase (GAPDH). Data are presented as mean relative expression values with the standard error of the mean (SEM). The sequences of the oligonucleotides used are as follows [13]:

GAPDH-F: CTGGGCTACACTGAGCACC

GAPDH-R: AAGTGGTCGTTGAGGGCAATG

SHANK2-F: CTTTGGATTCGTGCTTCGAG

SHANK2-R: CATCCACGGACTCCAGGTA

### 2.7. Western Blotting

Cells were lysed in RIPA buffer (Thermo Scientific) supplemented with protease and phosphatase inhibitors (Roche) to extract total protein. The protein concentration was determined using the BCA Protein Assay Kit (Pierce). Equal amounts of protein were separated by SDS-PAGE on a 10% polyacrylamide gel and transferred onto PVDF membranes (Millipore). The membranes were blocked with 5% nonfat dry milk in Tris-buffered saline containing 0.1% Tween-20 (TBST) for 1 h at room temperature. Following blocking, the membranes were incubated overnight at 4°C with primary antibodies against SHANK2 (dilution 1:1000) and beta-actin (dilution 1:5000) as a loading control. After washing with TBST, the membranes were incubated with HRP-conjugated secondary antibodies (dilution 1:2000; Cell Signaling Technology) for 1 h at room temperature. Protein bands were detected using an enhanced chemiluminescence (ECL) detection system (Thermo Scientific), and the signals were quantified using ImageJ software.

### 2.8. MTT Assay

Cell viability was assessed using the MTT assay. Transfected U87 and A172 cells were seeded in 96-well plates at a density of 3000 cells per well and allowed to adhere overnight. Then, 20 *μ*L of MTT solution (5 mg/mL in PBS) was added to each well at designated time points, and the plates were incubated for 4 h at 37°C. Following incubation, the medium was carefully removed, and 150 *μ*L of dimethyl sulfoxide was added to each well to dissolve the formazan crystals formed by metabolically active cells. The absorbance was measured at 570 nm using a microplate reader. Cell viability was expressed as a fold change relative to the control group.

### 2.9. Ethics

The information regarding normal brain tissue samples was sourced from healthy individuals in the GTEx database. Since our study utilized publicly available data from the GTEx and TCGA databases, our institutional ethics committee waived the need for additional approval.

## 3. Results

### 3.1. Link Between SHANK2 Expression Patterns and Molecular Characteristics of Glioma

In the study, we explored the correlations between SHANK2 expression levels and various patient characteristics in a cohort of 699 glioma cases (Table 1). The analysis revealed no significant gender-based differences in SHANK2 expression. However, age showed a strong correlation, with patients aged 60 years or younger more likely to exhibit high SHANK2 expression, a trend that was statistically significant (*p* < 0.001). Histological type and WHO grade also demonstrated significant associations with SHANK2 levels; notably, glioblastoma cases predominantly exhibited low SHANK2 expression, whereas oligodendrogliomas showed high expression levels. The genetic markers, including IDH status and 1p/19q codeletion, were significantly correlated with SHANK2 expression.

**Figure 1:**
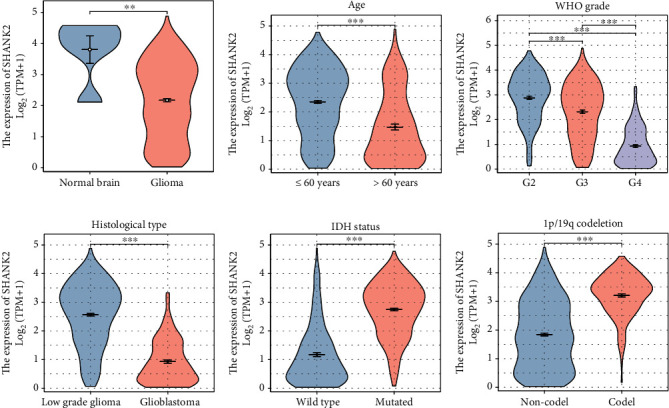
SHANK2 expression and its association with glioma characteristics. (a) Violin plot illustrating SHANK2 expression levels in normal brain tissue compared to glioma, indicating a significant increase in glioma (*p* < 0.01). (b) SHANK2 expression in patients stratified by age demonstrates a higher expression in patients aged 60 years and below (*p* < 0.001). (c) The relationship between SHANK2 expression and WHO grade of glioma, showing a stepwise increase in expression from Grades 2 to 3 and a decrease in Grade 4 (*p* < 0.001). (d) Comparison of SHANK2 expression in low-grade glioma versus glioblastoma, with low-grade glioma exhibiting higher levels (*p* < 0.001). (e) SHANK2 expression in glioma patients with wild-type versus mutated IDH status; higher expression is associated with mutated IDH (*p* < 0.001). (f) Analysis of SHANK2 expression with respect to the 1p/19q codeletion status shows higher expression in patients with codeletion (*p* < 0.001). The expression of SHANK2 is presented on a log2 scale, with each plot displaying the median and interquartile range.

For a more comprehensive visualization, we conducted the Kruskal–Wallis test regarding the SHANK2 expression in glioma, revealing distinct patterns across various clinical categories. In glioma tissues, SHANK2 is markedly elevated compared to normal brain tissue (Figure 1(a)), particularly in patients aged 60 and younger (Figure 1(b)). A gradation in SHANK2 levels is observed across different WHO grades, with a notable peak at Grade 2 and a subsequent decline in Grades 3–4 gliomas (Figure 1(c)). Interestingly, LGGs exhibit higher SHANK2 expression than glioblastomas (Figure 1(d)). Moreover, gliomas with mutated IDH status and those with 1p/19q codeletion are associated with higher SHANK2 expression, suggesting a potential link between SHANK2 and these genetic alterations (Figures 1(e) and 1(f)). These patterns underscore the potential role of SHANK2 expression as a differential marker in glioma biology and patient stratification.

### 3.2. High SHANK2 Expression is an Indicative Biomarker for Enhanced Glioma Survival

The detailed overall survival analysis in Table 2 showcases the prognostic significance of various factors in glioma patients through univariate analysis. Age above 60, histological type (notably glioblastoma), and genetic markers (IDH status and 1p/19q codeletion) were highlighted as significant prognostic factors, alongside SHANK2 expression levels. Specifically, age over 60 and glioblastoma presented with markedly increased HRs, indicating higher risks, while mutated IDH status and 1p/19q codeletion were associated with significantly reduced risks.

**Figure 2:**
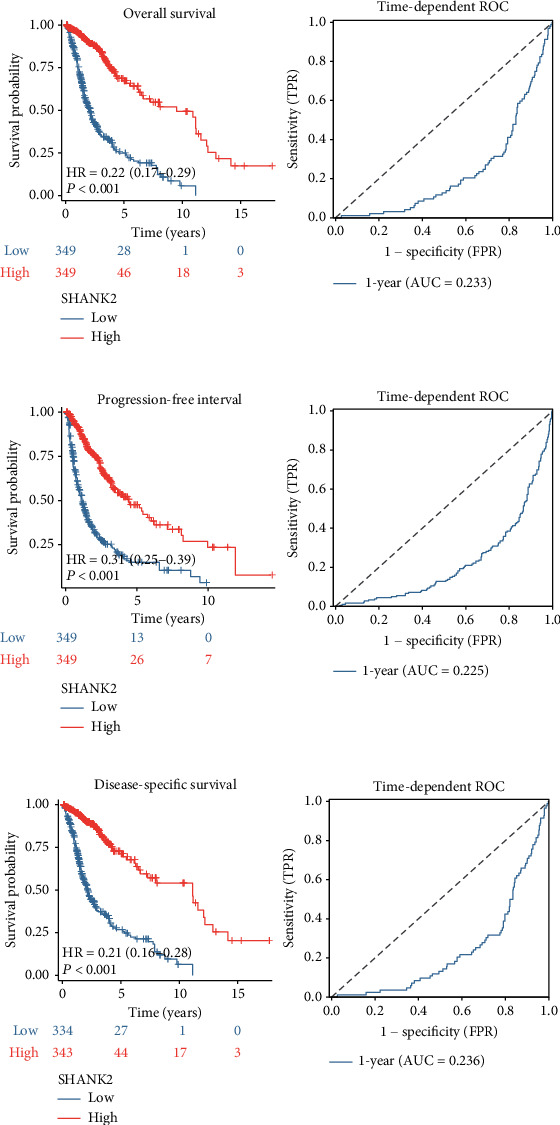
Survival analysis and diagnostic accuracy of SHANK2 expression in glioma. (a) Overall survival analysis using Kaplan–Meier curves for low versus high SHANK2 expression groups, showing significantly longer survival in the high SHANK2 group (HR = 0.22, *p* < 0.001). The right panel depicts a time-dependent ROC curve for 1-year survival, indicating the predictive performance of SHANK2 expression (AUC = 0.233). (b) Disease-specific survival analysis with Kaplan–Meier estimates for patients grouped by SHANK2 expression, with the high expression group demonstrating improved survival (HR = 0.21, *p* < 0.001). Time-dependent ROC analysis for 1-year survival is shown on the right (AUC = 0.236). (c) Kaplan–Meier curve showing progression-free survival of glioma patients with low versus high SHANK2 expression, revealing a significant survival advantage in the high expression group (HR = 0.34, *p* < 0.001). The corresponding time-dependent ROC curve for 1-year survival is presented (AUC = 0.225). Statistical significance was evaluated using Cox hazard regression tests, and hazard ratios were calculated with 95% CIs. Asterisks denote the level of significance, with ^∗∗∗^ indicating *p* < 0.001. The number of patients at risk at different time points is provided below each survival curve.

Of note, high SHANK2 expression was associated with a significantly reduced risk of poor outcomes, as indicated by a HR of 0.222, with a 95% CI, ranging from 0.167 to 0.294 in the univariate analysis (Figure 2(a), *p* < 0.001). Additionally, progression-free and disease-specific survival are both markedly better in patients with higher SHANK2 expression (Figures 2(b) and 2(c)). This is quantitatively underscored by HRs significantly less than 1, and modest yet consistent AUC values of 0.21–0.31 for 1-year survival predictions. These findings collectively affirm the potential of high SHANK2 expression as an indicative biomarker for enhanced survival in the glioma patient population.

We next conducted multivariate analyses by enrolling all the factors with *p* value less than 0.05 into the hazard regression model. As a result, patients older than 60 years had a notably increased risk of poor outcomes, with a HR of 1.531 (95% CI: 1.129–2.076, *p* = 0.006). Glioblastoma histological type was associated with a significantly elevated risk (HR = 2.308, CI: 1.573–3.388, *p* < 0.001), while oligoastrocytoma and oligodendroglioma did not significantly alter risk compared to astrocytoma. Conversely, mutated IDH status significantly reduced the risk of poor outcomes (HR = 0.256, CI: 0.176–0.373, *p* < 0.001). The presence of a 1p/19q codeletion did not significantly affect risk (HR = 0.789, CI: 0.451–1.382, *p* = 0.408). Importantly, high SHANK2 expression significantly and independently predicted better survival outcomes, with an HR of 0.650 (95% CI: 0.453 to 0.932; *p* = 0.019).

### 3.3. Stratification Analyses of the SHANK2's Prognostic Role

**Figure 3:**
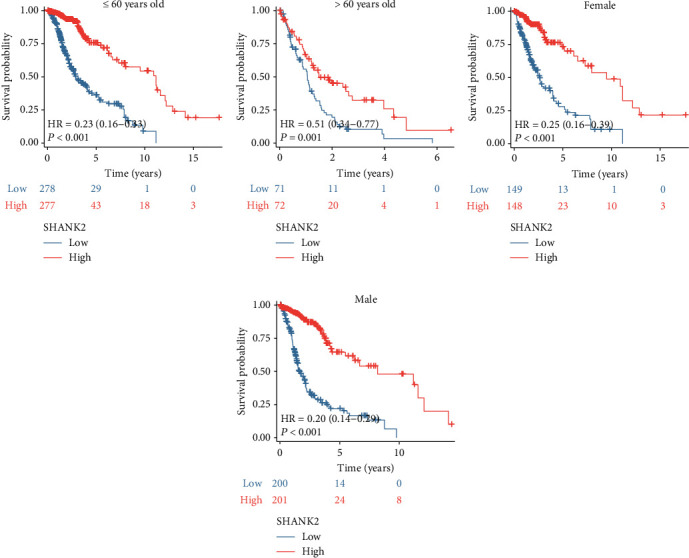
Impact of SHANK2 expression on survival based on age and gender in gliomas. (a) Kaplan–Meier survival curve for glioma patients ≤ 60 years old, showing significantly longer survival in those with high SHANK2 expression compared to low SHANK2 expression (HR = 0.23, *p* < 0.001). (b) In patients > 60 years old, high SHANK2 expression is also associated with improved survival, although the effect is less pronounced (HR = 0.51, *p* = 0.001). (c) Female patients with high SHANK2 expression exhibit a substantial survival benefit (HR = 0.25, *p* < 0.001). (d) Male patients demonstrate a similar trend, with high SHANK2 expression correlating with a marked increase in survival (HR = 0.20, *p* < 0.001).

Stratification analyses were further conducted to better illustrate the prognostic significance of SHANK2 in different subgroups. For example, patients under 60 exhibit markedly better survival rates with high SHANK2 expression (Figure 3(a), *p* < 0.001), a trend that persists, though less dramatically, in older patients (Figure 3(b), *p* = 0.001). Notably, both female and male patients with high SHANK2 levels show significant survival benefits (Figures 3(c) and 3(d), *p* < 0.001), suggesting the gene's expression confers a general survival advantage independent of gender and more pronounced in younger individuals.

**Figure 4:**
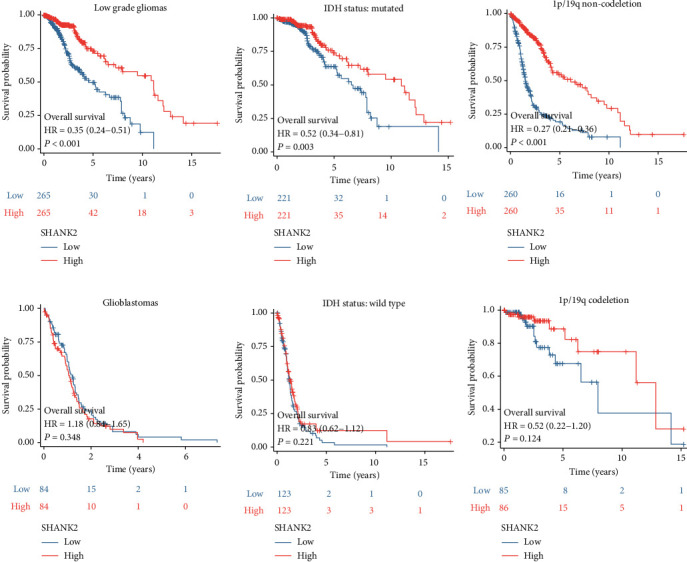
Survival analysis in glioma patients by SHANK2 expression and clinical features. (a) Kaplan–Meier curves for overall survival in low-grade glioma patients, stratified by SHANK2 expression levels. High SHANK2 expression correlates with significantly increased survival (HR = 0.35, *p* < 0.001). (b) Overall survival in patients with mutated IDH status, with high SHANK2 expression indicating improved survival (HR = 0.52, *p* = 0.003). (c) Overall survival in patients without 1p/19q codeletion, with high SHANK2 expression demonstrating a significant survival benefit (HR = 0.27, *p* < 0.001). (d) Kaplan–Meier curves for glioblastoma patients, showing no significant survival difference between low and high SHANK2 expression groups (HR = 1.18, *p* = 0.348). (e) Overall survival in patients with wild-type IDH status, showing no significant difference in survival based on SHANK2 expression (HR = 0.83, *p* = 0.221). (f) Kaplan–Meier curves for patients with 1p/19q codeletion, where SHANK2 expression does not significantly impact survival (HR = 0.52, *p* = 0.124).

Similarly, the survival outcomes for glioma patients, as stratified by SHANK2 expression and glioma characteristics, exhibit intriguing patterns. High SHANK2 expression correlates with enhanced survival in LGGs, IDH-mutated, and 1p/19q codeletion cases, emphasizing its potential as a favorable prognostic indicator (Figures 4(a), 4(b), and 4(c)). In stark contrast, SHANK2's expression level does not significantly influence survival in patients with glioblastomas or those with wild-type IDH status and 1p/19q codeletion (Figures 4(d), 4(e), and 4(f)). This dichotomy highlights SHANK2's expression as a differentially impactful factor in the complex landscape of glioma prognosis.

### 3.4. Survival Nomogram and Validation of Gliomas

**Figure 5:**
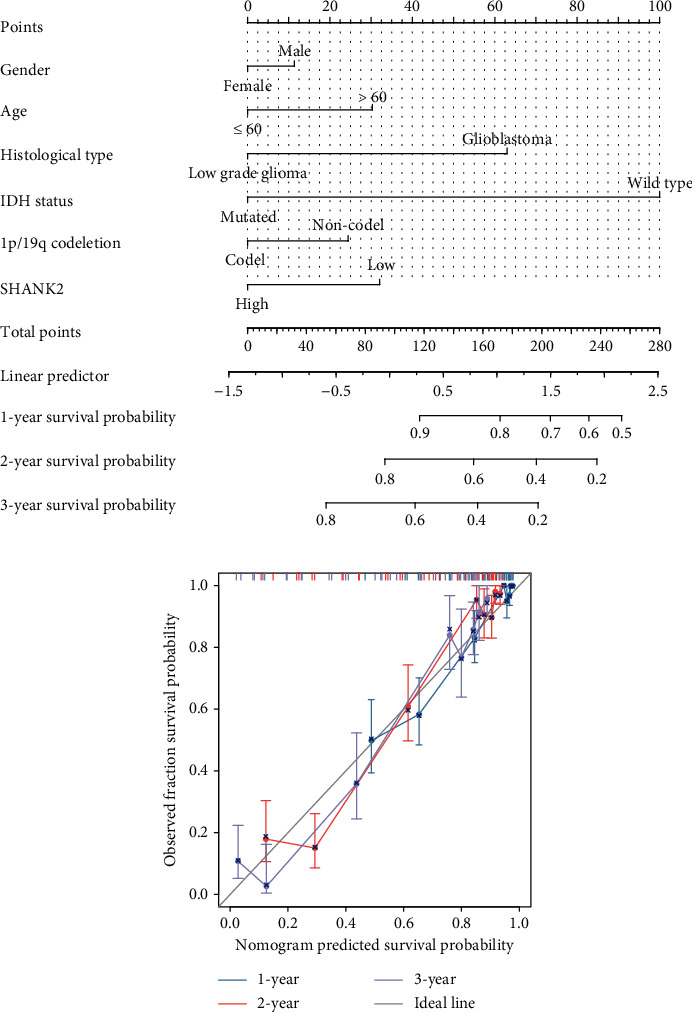
Prognostic nomogram for glioma patients and its validation. (a) A prognostic nomogram integrating patient gender, age, histological type, IDH status, 1p/19q codeletion status, and SHANK2 expression level. Each variable is allotted points which are totalled to predict the 1-, 2-, and 3-year survival probabilities for glioma patients. (b) Calibration plots compare the nomogram-predicted survival probability against the observed survival fraction at 1, 2, and 3 years. The closeness of the calibration plot points to the grey ideal line indicates the accuracy of the nomogram predictions.

Finally, we constructed a nomogram, a predictive tool that amalgamates various prognostic factors, such as gender, age, and histological features, along with SHANK2 expression levels, to estimate the survival probabilities for glioma patients (Figure 5(a)). Each factor is quantified with points, which are summed to predict survival at specific time intervals. The accompanying calibration plot assesses the nomogram's predictive accuracy by comparing expected to observed survival, with the ideal predictions lying on a designated line (Figure 5(b)). This nomogram is a visual representation that aids in predicting the probability of survival based on individual patient data and can be used to facilitate clinical decision-making.

### 3.5. Silencing SHANK2 Induces Glioma Cell Proliferation

**Figure 6:**
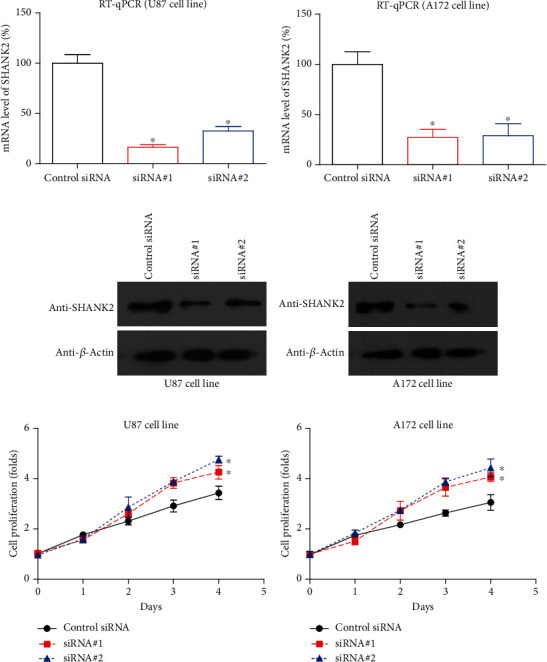
SHANK2 knockdown enhances cell proliferation in U87 and A172 glioma cell lines. (a, b) Relative mRNA expression levels of SHANK2 in U87 and A172 cells were measured by RT-qPCR following transfection with control siRNA or two distinct siRNAs targeting SHANK2. Both siRNAs significantly reduced SHANK2 mRNA levels compared to the control. (c, d) Western blot analysis confirmed the reduction of SHANK2 protein levels in U87 and A172 cells after siRNA-mediated knockdown, with beta-actin used as a loading control. (e, f) Cell proliferation was assessed over 4 days using the MTT assay. SHANK2 knockdown led to a significant increase in cell proliferation in both U87 and A172 cells, as compared to cells transfected with control siRNA. Data are presented as mean ± SEM. ^∗^*p* < 0.05 versus control siRNA.

Knockdown of SHANK2 in U87 and A172 glioma cell lines was confirmed by both RT-qPCR and Western blot analysis. The RT-qPCR results demonstrated a significant reduction in SHANK2 mRNA levels following transfection with two distinct siRNAs targeting SHANK2, compared to the control siRNA, in both U87 (Figure 6(a)) and A172 (Figure 6(b)) cell lines. This knockdown was further validated at the protein level, as Western blot analysis revealed a marked decrease in SHANK2 expression in both U87 (Figure 6(c)) and A172 (Figure 6(d)) cells transfected with SHANK2-specific siRNAs. Functionally, SHANK2 knockdown resulted in a significant increase in cell proliferation over a 4-day period, as shown in the MTT assay for both U87 (Figure 6(e)) and A172 (Figure 6(f)) cells. These findings suggest that SHANK2 acts as a suppressor of glioma cell proliferation.

## 4. Discussions

Glioma prognosis and treatment outcomes have long been subjects of intensive research, with genetic markers emerging as critical determinants of tumor behavior and patient survival. Studies have identified several key genes and molecular pathways that influence glioma pathogenesis and progression. Notably, mutations in the IDH1 and IDH2 genes are recognized for their role in lower-grade gliomas and secondary glioblastomas, offering prognostic and therapeutic implications [14]. The presence of MGMT (O^6^-methylguanine-DNA methyltransferase) promoter methylation has been correlated with better responses to alkylating agent chemotherapy, highlighting its value as a predictive marker for treatment planning [15].

Additionally, the 1p/19q codeletion has been associated with a favorable prognosis and increased sensitivity to chemotherapy and radiation in oligodendrogliomas, guiding therapeutic decisions [16]. These genetic insights have not only enhanced our understanding of glioma biology but also facilitated the development of more personalized treatment approaches. However, the heterogeneity of gliomas and their complex genetic landscapes continue to pose challenges in accurately predicting outcomes and tailoring therapies [17].

While the involvement of SHANK2 in various cancers is beginning to be elucidated, its role in glioma, particularly in influencing prognosis, remains largely unexplored [18]. The absence of comprehensive studies examining SHANK2 in glioma contexts suggests a crucial area for future research. Understanding whether SHANK2 expression levels correlate with tumor grade, patient survival, or treatment responsiveness could offer new avenues for prognostication and potentially identify novel therapeutic targets.

Our findings significantly enhance our understanding of the SHANK2 gene's role in glioma. Notably, SHANK2's higher expression levels correlating with increased survival rates introduce the possibility of a novel protective genetic factor within the glioma context, distinguishing it from more established prognostic markers. Despite these findings, the mechanisms by which SHANK2 influences cancer biology remain largely undefined. The mechanistic implications of SHANK2 in glioma are intriguing, considering its primary role in synaptic assembly and cognitive function [19]. It is possible that SHANK2's influence extends beyond the synapse, affecting glioma cell biology through alterations in intracellular signaling cascades, cellular adhesion, and migration patterns. These potential pathways may impact glioma cell proliferation and invasiveness, suggesting that SHANK2's role in the brain could be dual-faceted, encompassing both neuronal function and tumor suppression.

Furthermore, our findings pose questions about SHANK2's interaction with current therapeutic strategies for glioma. Does SHANK2 expression modulate sensitivity to chemotherapy or radiotherapy? Could it serve as a biomarker to guide the use of targeted therapies? These are critical questions for future clinical research. Our study is, however, limited by its reliance on retrospective data, which inherently carries the risk of confounding factors and biases. Prospective studies and clinical trials are essential to validate SHANK2's prognostic value. Moreover, the molecular mechanisms of SHANK2 in glioma remain unknown. Laboratory-based experiments are necessary to delineate the specific pathways through which SHANK2 exerts its effects on tumor cells.

One of the key limitations of this study is its retrospective design, which inherently introduces the risk of selection bias and confounding factors. Given that the data were sourced from multiple institutions, variations in patient management, diagnostic criteria, and treatment protocols could contribute to inconsistencies and biases in the analysis. The reliance on historical records also raises the possibility of incomplete or inconsistently documented data, which may affect the reliability of the findings. Moreover, the retrospective nature of the study limits our ability to establish causality, as the observed associations may be influenced by unmeasured confounding variables. The temporal span of the data adds another layer of complexity, as clinical practices and treatment guidelines may have evolved over the study period, potentially impacting the outcomes. Additionally, differences in follow-up duration and methodologies across the data sources could lead to variability in outcome measurements. To address these limitations, we employed rigorous data-cleaning processes and robust statistical methods to control for potential confounders and biases. Where feasible, we also conducted sensitivity analyses to validate our findings. Despite these efforts, it is important to acknowledge these limitations, and future prospective studies are needed to confirm and expand upon our results.

## 5. Conclusions

In conclusion, while our study presents a compelling case for SHANK2 as a prognostic marker in glioma, it is imperative to approach these findings with a view toward comprehensive validation and investigation. The potential for SHANK2 to influence glioma prognosis is clear, but the path from genetic marker to therapeutic target is a complex one that requires careful navigation through both clinical and molecular research landscapes.

## Figures and Tables

**Table 1:** Correlations between SHANK2 level and glioma patients' characteristics.

**Characteristics**	**Low expression of SHANK2**	**High expression of SHANK2**	**p** ** value**
Total cases	349	350	
Gender, *n* (%)			0.464
Female	144 (48.3%)	154 (51.7%)	
Male	205 (51.1%)	196 (48.9%)	
Age, *n* (%)			< 0.001
≤ 60 years old	242 (43.5%)	314 (56.5%)	
> 60 years old	107 (74.8%)	36 (25.2%)	
Histological type, *n* (%)			< 0.001
Astrocytoma	113 (57.7%)	83 (42.3%)	
Oligoastrocytoma	38 (28.1%)	97 (71.9%)	
Oligodendroglioma	39 (19.5%)	161 (80.5%)	
Glioblastoma	159 (94.6%)	9 (5.4%)	
WHO grade, *n* (%)			< 0.001
G2	59 (26.3%)	165 (73.7%)	
G3	109 (44.5%)	136 (55.5%)	
G4	159 (94.6%)	9 (5.4%)	
IDH status, *n* (%)			< 0.001
Wild type	210 (85.4%)	36 (14.6%)	
Mutated	131 (29.6%)	312 (70.4%)	
1p/19q codeletion, *n* (%)			< 0.001
Noncodel	324 (62.3%)	196 (37.7%)	
Codel	18 (10.5%)	154 (89.5%)	

Abbreviations: IDH, isocitrate dehydrogenase; WHO, World Health Organization.

**Table 2:** Univariate and multivariate Cox hazard regression analyses of glioma patients on overall survival.

**Characteristics**	**Total (** **N** **)**	**Univariate analysis**		**Multivariate analysis**	
		**Hazard ratio (95% CI)**	**p** ** value**	**Hazard ratio (95% CI)**	**p** ** value**
Gender	698				
Female	297	Reference			
Male	401	1.250 (0.979–1.595)	0.073		
Age	698				
≤60 years old	555	Reference		Reference	
>60 years old	143	4.696 (3.620–6.093)	< 0.001	1.531 (1.129–2.076)	0.006
Histological type	698				
Astrocytoma	196	Reference		Reference	
Oligoastrocytoma	135	0.646 (0.412–1.013)	0.057	0.953 (0.598–1.518)	0.839
Oligodendroglioma	199	0.578 (0.393–0.849)	0.005	0.843 (0.539–1.320)	0.456
Glioblastoma	168	6.791 (4.931–9.352)	< 0.001	2.308 (1.573–3.388)	< 0.001
IDH status	688				
Wild type	246	Reference		Reference	
Mutated	442	0.116 (0.089–0.151)	< 0.001	0.256 (0.176–0.373)	< 0.001
1p/19q codeletion	691				
Noncodel	520	Reference		Reference	
Codel	171	0.225 (0.147–0.346)	< 0.001	0.789 (0.451–1.382)	0.408
SHANK2 level	698				
Low	349	Reference		Reference	
High	349	0.222 (0.167–0.294)	< 0.001	0.650 (0.453–0.932)	0.019

Abbreviations: CI, confidence interval; IDH, isocitrate dehydrogenase; WHO, World Health Organization.

## Data Availability

Original data will be available upon reasonable request.
